# Genotyping *Plasmodium falciparum* gametocytes using amplicon deep sequencing

**DOI:** 10.1186/s12936-024-04920-3

**Published:** 2024-04-06

**Authors:** Jimmy Vareta, Natalie A. Horstman, Matthew Adams, Karl B. Seydel, Robert S. McCann, Lauren M. Cohee, Miriam K. Laufer, Shannon Takala-Harrison

**Affiliations:** 1 Center for Vaccine Development and Global Health, University of Maryland School of Medicine, Baltimore, MD USA; 2 Division of Cardiology, Department of Medicine, Johns Hopkins University School of Medicine, Baltimore, MD USA; 3 Blantyre Malaria Project, Kamuzu University of Health Sciences, Blantyre, Malawi; 4 College of Osteopathic Medicine, Michigan State University, East Lansing, MI USA

**Keywords:** *Plasmodium falciparum*, Complexity of infection, Gametocyte genotyping, Amplicon deep sequencing, Malaria transmission

## Abstract

**Background:**

Understanding the dynamics of gametocyte production in polyclonal *Plasmodium falciparum* infections requires a genotyping method that detects distinct gametocyte clones and estimates their relative frequencies. Here, a marker was identified and evaluated to genotype *P. falciparum* mature gametocytes using amplicon deep sequencing.

**Methods:**

A data set of polymorphic regions of the *P. falciparum* genome was mined to identify a gametocyte genotyping marker. To assess marker resolution, the number of unique haplotypes in the marker region was estimated from 95 Malawian *P. falciparum* whole genome sequences. Specificity of the marker for detection of mature gametocytes was evaluated using reverse transcription-polymerase chain reaction of RNA extracted from NF54 mature gametocytes and rings from a non-gametocyte-producing strain of *P. falciparum*. Amplicon deep sequencing was performed on experimental mixtures of mature gametocytes from two distinct parasite clones, as well as gametocyte-positive *P. falciparum* field isolates to evaluate the quantitative ability and determine the limit of detection of the genotyping approach.

**Results:**

A 400 bp region of the *pfs230* gene was identified as a gametocyte genotyping marker. A larger number of unique haplotypes was observed at the *pfs230* marker (34) compared to the *sera-2* (18) and *ama-1* (14) markers in field isolates from Malawi. RNA and DNA genotyping accurately estimated gametocyte and total parasite clone frequencies when evaluating agreement between expected and observed haplotype frequencies in gametocyte mixtures, with concordance correlation coefficients of 0.97 [95% CI: 0.92–0.99] and 0.92 [95% CI: 0.83–0.97], respectively. The detection limit of the genotyping method for male gametocytes was 0.41 *pfmget* transcripts/µl [95% CI: 0.28–0.72] and for female gametocytes was 1.98 *ccp4* transcripts/µl [95% CI: 1.35–3.68].

**Conclusions:**

A region of the *pfs230* gene was identified as a marker to genotype *P. falciparum* gametocytes. Amplicon deep sequencing of this marker can be used to estimate the number and relative frequency of parasite clones among mature gametocytes within *P. falciparum* infections. This gametocyte genotyping marker will be an important tool for studies aimed at understanding dynamics of gametocyte production in polyclonal *P. falciparum* infections.

**Supplementary Information:**

The online version contains supplementary material available at 10.1186/s12936-024-04920-3.

## Background

During the erythrocytic stage of the malaria parasite in humans, a fraction of asexual parasites differentiates into gametocytes [1]. Gametocytes are the sexual form of the parasite transmitted from humans to mosquitoes and are an important target for interventions that interrupt human-to-mosquito transmission [1, 2]. Polyclonal *Plasmodium falciparum* infections are prevalent in high transmission settings [3–6]. As multiple different asexual parasite clones are circulating and multiplying in the peripheral bloodstream, the contribution of each clone to the total gametocytaemia and the factors that impact the relative gametocytaemia of different clones are not clear. Understanding the dynamics of gametocytaemia corresponding to different clones in the context of polyclonal *P. falciparum* infections will help identify parasite and/or host factors that influence gametocyte production during natural infection. Such knowledge will fill a gap in the current understanding of gametocyte biology, which may inform development of malaria prevention strategies targeting human-to-mosquito transmission of the parasite.

Identifying the specific parasite clones that have produced gametocytes in a polyclonal *P. falciparum* infection requires a genotyping assay that distinguishes clones in both asexual parasites and mature gametocytes. Development of such an assay requires identification of a polymorphic region of a gene primarily expressed in mature gametocytes to serve as a genotyping marker. Identification of gametocyte-specific markers for genotyping has been challenging, as many genes are not exclusively expressed in gametocytes [7], and those that are, often display relatively limited genetic diversity [8]. Genotyping of gametocyte-specific markers will allow estimation of clone composition among gametocytes (by genotyping RNA) and all parasite blood stages (by genotyping genomic DNA) in polyclonal *P. falciparum* infections. Previous gametocyte genotyping methods have utilized length-polymorphism markers in genes encoding proteins such as *osmiophilic body protein* (*pfg377*) and *6-cysteine protein P230* (*pfs230)* [9–13]. Such length-polymorphism markers are prone to amplification bias towards smaller fragments and rely on gel or capillary electrophoresis to determine fragment sizes, resulting in limited sensitivity, low throughput and resolution, and inability to quantify relative clone frequencies [14–16].

With increased access to short-read sequencing technologies, researchers are performing deep sequencing of small amplicons representing regions of high heterozygosity in *P. falciparum* antigen genes to estimate infection complexity and parasite genetic relatedness, and to track malaria transmission dynamics [4, 14, 16–21]. The large read depth of amplicon deep sequencing allows for an increased ability to detect low frequency clones (as low as 1%) and quantification of relative clone frequencies in an infection based on the proportion of reads corresponding to a given haplotype [16, 22]. Furthermore, multiplexed sequencing increases throughput with the capability of sequencing up to 384 samples in one sequencing run using the Illumina MiSeq platform. Although amplicon deep sequencing is now readily used to genotype *P. falciparum* infections in field settings, it has not been adapted to genotype gametocytes in *P. falciparum* infections. Therefore, this study was designed to develop an amplicon deep sequencing approach to genotype *P. falciparum* mature gametocytes in natural infections.

## Methods

### Identification of the gametocyte genotyping marker

Genes expressed primarily in *P. falciparum* mature gametocytes were identified based on stage-specific single cell RNA-sequencing data [7]. Polymorphic regions of these genes were identified by mining a published *P. falciparum* microhaplotype data set containing 4,465 highly polymorphic genomic regions of 200 bp in length [23] for characterization as potential candidate gametocyte genotyping markers. To improve genotyping resolution, overlapping microhaplotype regions were combined (when possible) to obtain larger amplicons of a size amenable to coverage by Illumina MiSeq paired-end reads. The most diverse amplicon was selected as the candidate gametocyte genotyping marker. The genomic locations and expected heterozygosity of the three combined microhaplotypes comprising the genotyping marker are shown in Additional file 1: Table S1. Based on > 3800 global *P. falciparum* isolates, the three microhaplotype regions comprising the genotyping marker all had an expected heterozygosity > 50% and 9, 8, and 2 SNPs detected in each 200 bp region, respectively (Additional file 1: Table S1). To evaluate potential haplotype resolution of the candidate genotyping marker, the number of SNPs and unique haplotypes was estimated at the marker locus from whole genome sequence (WGS) data generated from 95 Malawian *P. falciparum* field isolates [24, 25] (average read depth =  ~ 150x). For comparison, the number of unique haplotypes at two loci widely used for blood stage amplicon sequencing (*ama-1* and *sera-2*) [26, 27] was also estimated from the same data set.

### Specificity of the candidate genotyping marker for detection of mature gametocytes

To determine the specificity of the candidate gametocyte genotyping marker for detection of mature gametocytes (compared to asexual parasite stages), RNA was extracted from cultured ring stage parasites (10^5^ rings/µL) of the MRA–1183 *P. falciparum* SenTh026.04 strain (a non-gametocyte producing line obtained from BEI Resources) [28] and mature gametocytes (10 gametocytes/µL) from the NF54 strain (a gametocyte-producing laboratory strain) using the RNeasy Mini kit (Qiagen, CA). Complementary DNA (cDNA) was synthesized from RNA using the QuantiTect Reverse Transcription kit (Qiagen, CA). PCR with primers targeting the candidate marker was performed on the cDNA using the KAPA HiFi HotStart ReadyMix PCR kit (Kapa Biosystems, Wilmington, MA). Primers were designed using Primer-BLAST [29] (Additional file 1: Table S2). A higher concentration of MRA-1183 rings compared to NF54 gametocytes was used to reflect the higher asexual parasite density (compared to gametocyte density) often observed in natural *P. falciparum* infections [30], and to determine if any low-level expression of the candidate marker gene in rings could overestimate mature gametocyte genotypes. A region of the *pfs25* gene, a gametocyte detection marker known to be highly specific to female gametocytes [31], was amplified as a positive control for gametocyte specificity. All RT-PCRs were done with a reverse-transcriptase negative control to rule out DNA contamination in extracted RNA. PCR products from the RT-PCR were visualized using agarose gel electrophoresis.

### Quantitative ability of the genotyping assay in experimental mixtures of gametocytes from distinct *P. falciparum* isolates

#### Mature gametocyte mixtures

Two distinct strains of *P. falciparum* (a Malawian field isolate (MLW5) and the NF54 strain) were cultured to obtain mature gametocytes. These strains differed at 2 sites within the length of the candidate genotyping marker sequence. Mature gametocytes from each of the two parasite strains were counted and mixed with uninfected human whole blood to obtain a stock of 100 gametocytes/µL. RNA was extracted from mature gametocytes from each strain stock using the extraction method described above. Genomic DNA was also extracted from the same gametocyte stocks using a QIAamp DNA blood kit (Qiagen, CA). RNA extracted from the mature gametocyte stocks from each of the two strains were mixed in duplicate in different proportions (NF54: MLW5 = 1:0, 10:1, 5:1, 2:1, 1:1, 1:2, 1:5, 1:10, and 0:1) to mimic RNA extracted from an infection with two unique gametocyte genotypes. DNA extracted from mature gametocyte stocks of the same two strains was also mixed at the same proportions described for the RNA mixtures.

#### Sequencing library preparation

A primary PCR was performed on cDNA from the mixtures of mature gametocytes using primers (including 5’ overhang sequences) targeting the gametocyte genotyping marker. A reverse transcriptase negative control and PCR negative and positive controls were included in all PCR runs. To allow multiplexing by sample, a secondary PCR (i.e., index PCR) was performed with primers targeting overhang sequences on primary PCR primers to attach unique barcodes and sequencing adapters (IDT for Illumina Nextera DNA UD Indexes Set A, B, C and D, San Diego, CA) (Additional file 1: Fig. S1). The primary RT–PCR and index PCR primer sequences, reaction mixes, and cycling conditions are provided in Additional file 1: Tables S2 and S3. Pooled barcoded amplicons were purified using the AMPure XP bead kit (Beckman, IN) and underwent sequencing using 300 bp paired-end reads on an Illumina MiSeq (Illumina). Sequencing runs included an Enterobacteria phage PhiX control (Illumina, PhiX Control v3) for quality control. The same primary and index PCR was performed on genomic DNA extracted from mature gametocyte mixtures.

#### Processing of sequencing reads

The SeekDeep [21] software package with default settings was used to demultiplex sequencing reads; trim adapter, overhang, primer and barcode sequences; assess read quality and remove poor quality reads; and call haplotypes. Trimmed paired-end reads were merged and clustered based on unique haplotypes after excluding likely chimeric reads and reads with insertions and deletions. Each unique haplotype generated from RT-PCR amplicons represents a distinct gametocyte clone and each unique haplotype generated from PCR amplicons represents a distinct clone amongst all sampled parasite life stages within the infection (in this case the experimental gametocyte mixture). Clone frequency was estimated based on the proportion of sequencing reads corresponding to a given haplotype out of the total number of reads from all clones in the infection/mixture.

### Limit of detection of the genotyping marker

#### Study samples

Samples for determination of the limit of detection (LOD) of the genotyping assay were obtained from a household-based cohort study conducted as part of the Malawi International Center of Excellence for Malaria Research (ICEMR). The study was conducted within the catchment areas of the Namanolo and Ntaja health centres in Balaka and Machinga districts, respectively, in southern Malawi from April 2019 to March 2020. The cohort study included 96 households, with all members of each household invited to participate in the study. Consent was sought from the head of each household, followed by individual level consent from other adults and assent from children > 14 years of age. The cohort included 959 individuals who were followed actively every 4–6 weeks, yielding a maximum of ten visits over the 10-month study. Passive case detection was conducted by a study nurse at the health centre, and malaria treatment was provided by health centre staff according to national guidelines. Blood samples were collected by finger prick at each active and passive case detection visit, with ~ 50 µL blotted onto filter paper (stored at room temperature with desiccant) for PCR detection of *P. falciparum* and 100 µL preserved in RNA Protect cell reagent (Qiagen, CA) (stored at -80 °C) for gametocyte detection.

#### Gametocyte detection

Gametocyte quantification for *P. falciparum*-positive samples was based on expression of the *ccp4* (female gametocyte) and *pfmget* (male gametocyte) genes, assessed using RT-qPCR as described previously [32]. In brief, RNA was extracted from each sample using the RNeasy Plus 96 kit (Qiagen). Five µL of RNA, and serially diluted standards, were used in a multiplex one-step RT-qPCR using the Luna Universal Probe One-Step RT-qPCR Kit (NEB) and probes and run on a Light Cycler 96 Real-time PCR machine (Roche). Standards were generated from target RNA copies transcribed in vitro using the MEGAShortscript T7 high yield transcription kit (Invitrogen) and were quantified using a Qubit 3 fluorometer (ThermoFisher). All RT-qPCR reactions were performed in duplicate, with a third PCR run as a tie breaker to resolve discordant results. Transcript abundance (estimated as transcripts/µL) for each gene in study samples was estimated using the average cycle threshold (Ct) of each marker and a linear regression of Ct values of the serially diluted standards and their respective transcripts/µL.

A total of 101 *P. falciparum* gametocyte-positive blood samples were selected to represent a range of *pfmget* and *ccp4* transcript abundance values for determination of the limit of detection (LOD) of the genotyping assay. RT-PCR targeting the candidate gametocyte genotyping marker was performed on RNA extracted from the gametocyte-positive samples and amplicons were sequenced as described above.

### Genotyping of DNA and RNA in field samples

The genotyping assay was applied to genotype DNA and RNA from 594 gametocyte-positive infections from the ICEMR cohort study (described above) to compare parasite haplotypes present in DNA (all circulating parasite stages) and RNA (circulating gametocytes) from the same infections. From among these infections, 209 RNA samples were successfully genotyped and 161 had corresponding DNA genotypes from the same infection, for a total of 161 paired DNA and RNA samples corresponding to 102 individuals.

### Data analysis

To evaluate whether the genotyping approach can estimate gametocyte clone frequency in a quantitative manner, a concordance correlation coefficient (CCC) [33] was used to estimate the agreement between observed haplotype frequencies in gametocyte mixtures after sequencing and expected haplotype frequencies based on ratios used to prepare mature gametocyte mixtures. A probit regression model was used to determine the limit of detection (LOD) of the gametocyte genotyping assay in field isolates by estimating the *pfmget* and *ccp4* transcripts/µL at which the probability of a sample being successfully genotyped is 0.95. Statistical analyses were performed using SAS 9.4 (SAS Institute Inc., Cary, North Carolina) and R (version 4.2.3).

## Results

### Selection of the gametocyte genotyping marker and its specificity to mature gametocytes

**Fig. 1:**
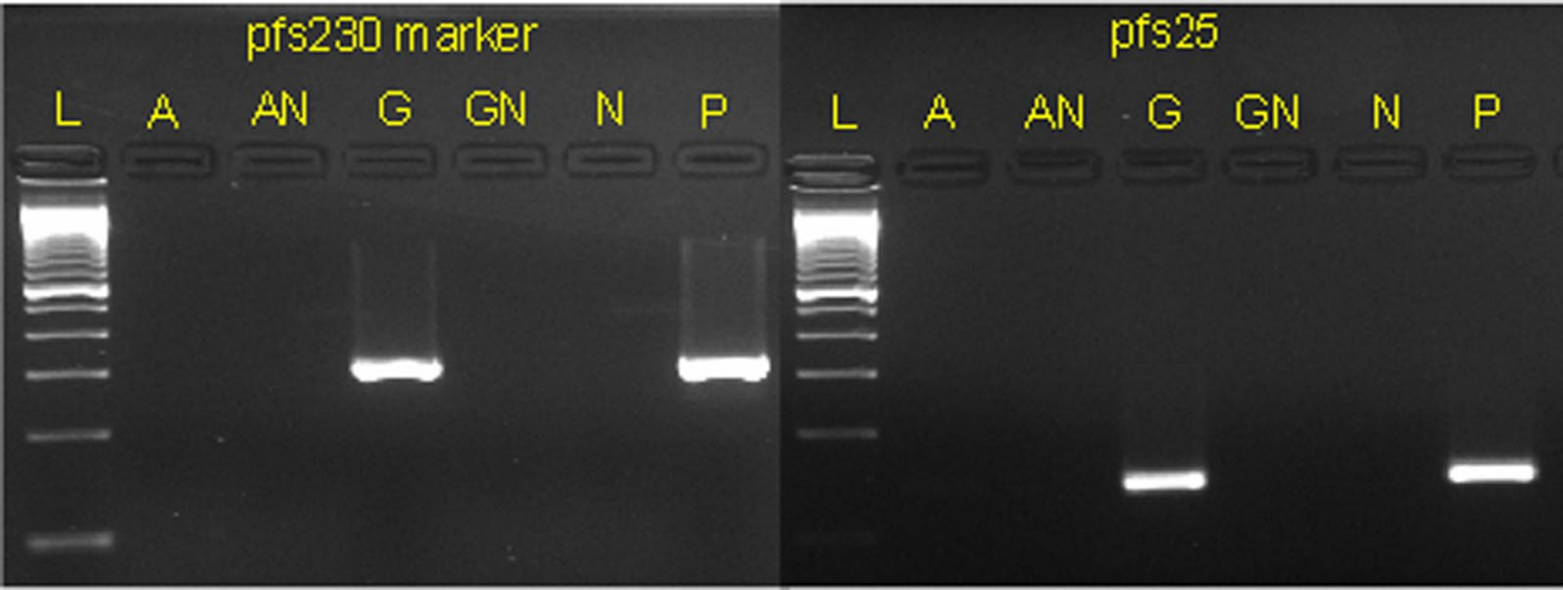
Specificity of the *pfs230* genotyping marker for detection of mature gametocytes. Lane A in each panel represents RT-PCR performed on RNA extracted from a high parasite density culture (100,000 parasites/µL) of a non-gametocyte-producing *P. falciparum* strain MRA-1183, with corresponding RT negative control (AN). Lane G represents the same RT-PCRs on RNA extracted from NF54 gametocytes (10 gametocytes/µL) with corresponding RT negative (GN) control. Lane N and P represent the PCR negative and positive controls, respectively. Lane L represents a 100bp DNA ladder

A 400 bp polymorphic region of the gene encoding *pfs230* (Pf3D7_0209000; coordinates: 375,686–376,086) was selected as a candidate gametocyte genotyping marker. The *pfs230* gene is expressed in both female and male gametocytes in *P. falciparum,* with greater expression in males than females [34]. The number of unique haplotypes observed at this locus, as estimated from WGS data generated from 95 Malawian *P. falciparum* isolates, was 34, while 18 and 14 unique haplotypes were observed at the *sera-2* and *ama-1* marker loci, respectively (Table 1). RT–PCR of the candidate marker in NF54 mature gametocytes and MRA–1183 *P. falciparum* SenTh026.04 ring stage parasites showed amplification of NF54 mature gametocytes and no amplification of MRA-1183 ring stage parasites, despite ring stage parasitaemia being four orders of magnitude greater than the gametocytaemia in the test sample, suggesting that the marker is specific to mature gametocytes (Fig. 1).

**Table 1:** Number of SNPs and unique haplotypes detected by the gametocyte genotyping marker (*pfs230*) and commonly used blood stage short amplicon markers (*sera-2* and *ama-1*) in whole genome sequences from 95 *P. falciparum* field isolates collected in Malawi

Gene ID	Gene Name	Marker Start	Marker End	SNPs	Number of haplotypes	Marker Size
Pf3D7_0209000	*pfs230*	375,686	376,086	11	34	400
Pf3D7_0207900	*sera-2*	320,763	321,020	5	18	257
Pf3D7_1133400	*ama-1*	1,294,056	1,294,456	5	14	400

### Estimating clone frequency among gametocytes and total parasites

**Fig. 2:**
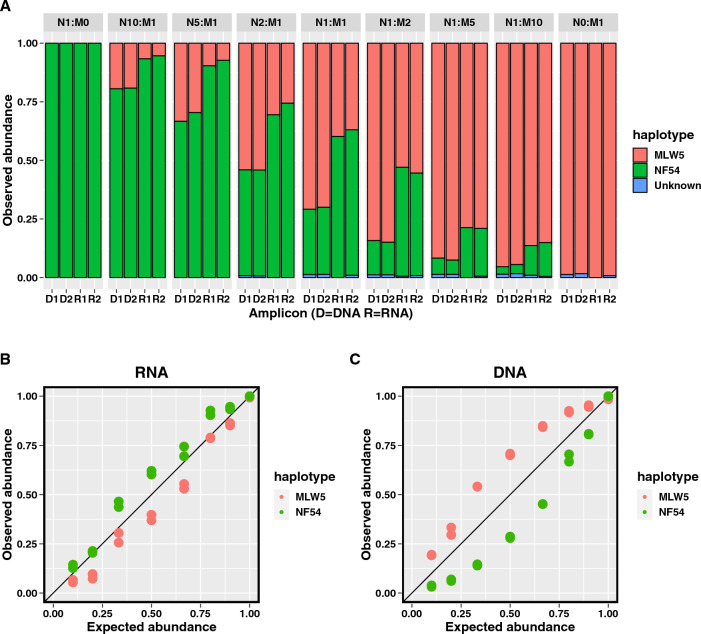
Agreement between expected clone abundance (gametocyte mixture ratios) and observed abundance (clone frequency as estimated by the proportion of reads corresponding to a given clone) of RNA and DNA extracted from NF54 and MLW5 mature gametocyte mixtures. **A** Stacked bar graphs depicting observed clone frequency after amplicon sequencing of mature gametocyte mixtures of NF54 (N) and MLW5 (M) strains DNA (D1 and D2 are replicates) and RNA (R1 and R2 are replicates) were mixed in the following ratios: 1:0, 10:1, 5:1, 2:1, 1:1, and the reverse. **B**, **C** The observed RNA and DNA clone frequencies for each clone are plotted against the expected frequencies (black diagonal line) and were found to be highly concordant (RNA CCC = 0.97; DNA CCC = 0.92)

The *pfs230* genotyping marker locus was amplified from DNA and RNA extracted from mature gametocyte mixtures, and the amplicons were sequenced. Sequencing coverage ranged from 4000 to 163,316 reads prior to read filtering. After filtering erroneous reads, sequencing coverage ranged from 2,819 to 37,445 reads. The observed proportions of reads corresponding to the two isolates in the DNA and RNA gametocyte mixtures are shown in Fig. 2A. The expected proportion of reads (proportions used to prepare gametocyte mixtures) for each isolate in the mixtures was compared to its corresponding observed proportion of reads. Agreement between expected and observed haplotype frequencies was high in both RNA (CCC = 0.97;95% CI: 0.92–0.99) and DNA (CCC = 0.92;95% CI: 0.83–0.97) mixtures (Fig. 2B and C). These results suggest that the genotyping assay accurately estimates relative clone frequency of gametocytes (when using RNA) and total parasite clone frequency (when using DNA) in polyclonal infections. A minority clone was detected in the Malawian field isolate that was previously undetected in the whole genome sequencing data generated from this isolate, a further testament of the increased sensitivity of amplicon deep sequencing for detection of minority clones (unknown haplotype in Fig. 2A).

### Limit of detection of the genotyping assay

**Fig. 3:**
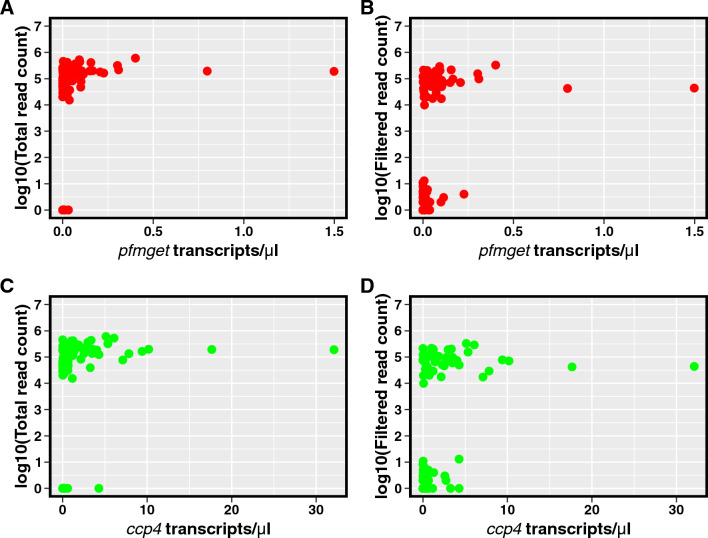
Scatter plots of total and filtered reads by *pfmget* (male gametocyte) and *ccp4* (female gametocyte) transcript abundance. **A**, **B** Scatter plots of raw and filtered read count by *pfmget* transcripts/µL. **C**, **D** Scatter plots of raw and filtered read count by *ccp4* transcripts/µL

To determine the LOD of the genotyping assay, cDNA from 101 gametocyte-positive samples underwent PCR followed by sequencing. After filtering reads using SeekDeep, two populations of reads were observed: one ranging from 0 to 15 per sample and another ranging from 10,000 to 100,000 per sample (Fig. 3). Samples with filtered reads ranging from 0 to 15 were called genotyping failures and samples with filtered reads ranging from 10,000 to 100,000 were considered successfully genotyped. The distribution of total and filtered read counts by female and male gametocyte marker transcript abundance are shown in Fig. 3A–D.

Out of 101 RNA samples sequenced, 48 (47%) were successfully genotyped. Fourteen unique haplotypes were observed among the 48 successfully genotyped samples: 27 samples (26.7%) with a single gametocyte haplotype; 18 (17.8%) with 2 gametocyte haplotypes; and 3 (3%) with 3 gametocyte haplotypes. Median *pfmget* transcript abundance in samples that were successfully genotyped was 0.17 transcripts/µL [IQR: 0.05–0.26] compared to 0.005 transcripts/µL [IQR: 0.002–0.02] in samples that failed sequencing. Median *ccp4* transcript abundance in samples that were successfully genotyped was 0.57 transcripts/µL [IQR: 0.16–1.43] compared to 0.03 transcripts/µL [IQR: 0.003–0.15] in samples that failed sequencing. Using probit regression, the LOD of the genotyping method based on the *pfmget* gene was estimated to be 0.41 transcripts/µL [95% CI: 0.28–0.72] and based on the *ccp4* gene, 1.98 transcripts/µL [95% CI: 1.35–3.68].

### Concordance of haplotypes between DNA and RNA genotyping

**Fig. 4:**
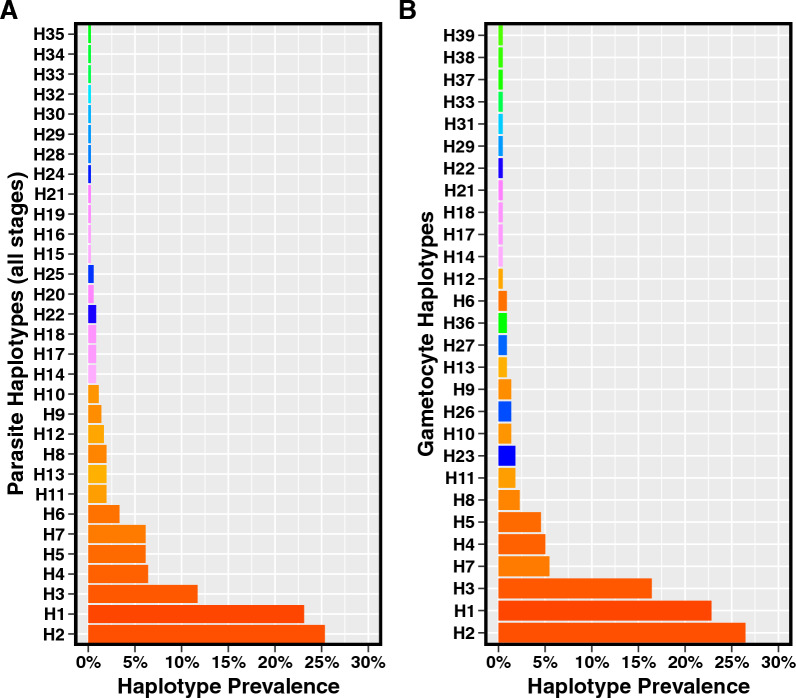
Frequency of unique haplotypes in 161 gametocyte-positive *P. falciparum* infections among **A** all parasite stages in the infection (DNA samples) and **B** gametocytes in the infection (RNA samples). Haplotypes are shown on the y-axis, with each haplotype represented by a unique-colored bar. The prevalence of the haplotype among the samples is shown on the x-axis

Amplicon sequencing with the *pfs230* marker was performed on DNA and RNA extracted from 594 gametocyte-positive *P. falciparum* infections from the ICEMR cohort study to determine the haplotypes present among all circulating parasite stages (DNA genotyping) and among circulating gametocytes (RNA genotyping) within each infection. From these infections, 209 RNA samples were successfully genotyped, with successful amplicon generation consistent with the estimated LOD of the assay (Additional file 1: Table S4), and 161 infections had both RNA and DNA genotypes available. Eighty-eight (54.7%) of the 161 infections were polyclonal. Thirty-nine unique haplotypes were observed among the RNA and DNA genotypes. Fifteen infections (9.3%) had a discordant haplotype identified in the RNA sample that was not observed in its paired DNA sample. The mean COI among all parasite stages (DNA samples) was 3.36 [range:1—8] and in gametocytes (RNA samples) was 1.67 [range:1–4] (Table 2). The prevalence of each haplotype was similar in DNA and RNA samples (Fig. 4), with the three most prevalent haplotypes being the same between all parasites (DNA) and gametocytes (RNA).

**Table 2:** Performance of the gametocyte genotyping assay in 161 *P. falciparum* gametocyte-positive paired DNA and RNA field samples

	Paired DNA/RNA samples (n = 161 infections, 102 individuals)
Unique haplotypes in all samples (DNA and RNA)	39
Unique haplotypes among all parasite stages (DNA samples)	31
Unique haplotypes among gametocytes (RNA samples)	28
Polyclonal infections	88 (54.7%)
Infections with an RNA haplotype not observed among DNA haplotypes	15 (9.3%)
Mean COI among all parasite stages (DNA samples)	3.36 [range: 1–8]
Mean COI among gametocytes	1.67 [range: 1–4]

## Discussion

In this study, an assay was developed to genotype mature gametocytes in *P. falciparum* infections through deep sequencing of RT-PCR amplicons generated from a polymorphic region of the *pfs230* gene. The genotyping assay is specific to mature gametocytes, is quantitative, and has higher haplotype resolution than known *P. falciparum* blood stage amplicon-based genotyping markers. The approach provides a useful tool to advance the understanding of how infection complexity and clone composition impact gametocyte production in *P. falciparum* infections. This knowledge will provide further insights into parasite transmission biology, which may inform development of transmission-interrupting interventions.

Notwithstanding the limited genetic diversity of many genes highly expressed in mature gametocytes [8], a polymorphic region of the *pfs230* gene was identified as a marker to genotype gametocytes in *P. falciparum* infections. Since the genotyping marker was selected from a published data set of microhaplotypes extracted from WGS data from *P. falciparum* isolates representative of global malaria isolates [23], gametocyte genotyping using this marker should be applicable in a range of malaria-endemic settings. The specificity of the genotyping marker to mature gametocytes was an important consideration in the assay development. Most genes that are highly expressed in mature gametocytes are also expressed at low levels in other asexual parasite stages, including ring stages [7]. Therefore, low-level expression of a marker gene in ring stage parasites, if detectable by RT-PCR, may overestimate the number of clones represented among gametocytes (because clones present among asexual stages will also be detected), especially in infections with high parasitaemia [32]. Here, the RT-PCR results showed that even at high ring stage parasitaemia (10^5^ rings/µL), there was no detectable expression of the marker gene. This finding demonstrates that the gametocyte genotyping marker is specific to mature gametocytes.

Because of high read depth when using amplicon deep sequencing (read depth range was 2,819 to 37,445 reads), the ability to detect minor clones in polyclonal infections is improved [22]. High concordance (CCC > 0.90) between observed and expected clone proportions for both RNA and DNA in the gametocyte mixtures suggest that the sequencing read depth was large enough to detect minor clones with frequency of 10% (the smallest dilution tested). The lowest detectable frequency of minor clones is likely < 10% based on the ability to detect a minor clone within the Malawian field isolate included in the experimental mixtures that has a frequency of < 2% and was not detected using WGS data with lower average read depth (~ 150x). The ability to generate quantitative estimates of parasite clone frequency among blood stage parasites and gametocytes within an infection may allow assessment of associations between parasite clone frequency (e.g., whether a given clone is a majority or minority clone within an infection) and parasite investment in sexual reproduction (although the contribution of gametocytes to total blood stage clone frequencies may need to be accounted for in such analyses). Furthermore, the ability to detect unique clones and their relative frequency could also allow identification of specific clones that more often produce gametocytes. Such parasites could then be characterized further to understand genetic determinants of increased gametocyte production.

A major challenge of genotyping gametocytes in field settings is that most infections have low gametocyte density, which can affect the number of samples that are successfully genotyped and the detection of minority clones in polyclonal infections [35]. This problem is compounded in submicroscopic infections, which are prevalent in many malaria endemic settings [36]. As noted, less than half of the gametocyte-positive field samples in the study were successfully genotyped. Gametocyte-positivity of the field samples was determined using a highly sensitive RT-qPCR [32]. The results suggest that this RT-qPCR is more sensitive than the PCR used to generate the *pfs230* amplicons for the genotyping assay, a finding similar to that observed for other gametocyte genotyping markers [10]. The proportion of field samples from which the *pfs230* amplicon could be successfully amplified and genotyped is consistent with the LOD of the assay, with the median *ccp4* and *pfmget* transcript abundance for all gametocyte-positive field samples being lower than the estimated LOD. Prior gene expression studies have indicated a fivefold higher expression of the *pfs230* gene in male gametocytes than in female gametocytes [34]. However, *P. falciparum* infections are often female-biased [1, 37], with a ratio of female to male gametocytes on the order of 2–5 females to 1 male or even higher depending on transmission intensity [38, 39]. The fact that many *P. falciparum* infections contain more female gametocytes may offset, to an extent, the lower expression of the genotyping marker gene in female gametocytes; however, where possible, downstream analyses should adjust results by gametocyte sex ratio.

DNA and RNA extracted from the same infections were genotyped to determine if observed haplotypes among all sampled parasite stages (DNA genotyping) are concordant with haplotypes observed among gametocytes (RNA genotyping). In a small proportion of infections (9.3%), haplotypes were observed in the RNA fraction (representing gametocytes) that were not observed in the DNA fraction (representing all parasites in the infection). This type of discordance has also been observed with other gametocyte genotyping markers, but in a 4–6 times larger proportion of infections than observed in this study, depending on the marker [10]. This discordance may reflect infections where gametocytes make up a comparatively small fraction of the total parasites and are below the limit of detection in the context of the overall infection represented by the DNA fraction.

## Conclusions

In this study, a gametocyte genotyping assay was developed based on deep sequencing of an amplicon marker within the *pfs230* gene. This high-resolution genotyping approach can detect minority clones within *P. falciparum* infections and can estimate relative clone frequencies. This genotyping assay can be used to answer important questions about parasite transmission biology, including the influence of polyclonal infections on gametocyte production and dynamics of gametocytes within *P. falciparum* infections.

### Supplementary Information

**Additional file 1: Table S1. **Microhaplotypes^**a**^ comprising the *pfs230 *gametocyte genotyping marker. **Table S2**: RT-PCR and PCR primer sequences for *pfs230*-M3A genotyping marker with overhang adapter sequences for library preparation. **Figure S1. **Two-step PCR to generate amplicons for sequencing. The first PCR targets the genotyping marker sequence, and primers contain overhang sequences at their 5` ends. The second PCR attaches sequencing adapters and unique indexes to the amplicons from the first PCR using primers with sequence complementary to the overhang sequences included in the primers for the first PCR. **Table S3: **Reverse transcription, PCR and index PCR reaction preparation and cycling conditions. **Table S4. ***ccp4 *and *pfmget *transcript abundance in samples collected from gametocyte-positive infections occurring in participants in a cohort study conducted in Malawi.

## Data Availability

Amplicon sequence data are available in the NCBI Sequence Read Archive (SRA) (Bioproject: PRJNA1048473).

## Abbreviations

CCC: Concordance correlation coefficient
cDNA: Complementary deoxyribonucleic acid
CI: Confidence interval
DNA: Deoxyribonucleic acid
IQR: Interquartile range
LOD: Limit of detection
RNA: Ribonucleic acid
RT-PCR: Reverse transcription polymerase chain reaction
PCR: Polymerase chain reaction
