# Supplementary material for: Genotyping Plasmodium falciparum gametocytes using amplicon deep sequencing
Source: Malar J. 2024 Apr 6;23:96. doi: 10.1186/s12936-024-04920-3 (PMC10999092; doi:10.1186/s12936-024-04920-3)
Supplement: Supplementary file 1 — Additional file 1: Table S1. Microhaplotypesa comprising the pfs230 gametocyte genotyping marker. Table S2: RT-PCR and PCR primer sequences for pfs230-M3A genotyping marker with overhang adapter sequences for library preparation. Figure S1. Two-step PCR to generate amplicons for sequencing. The first PCR targets the genotyping marker sequence, and primers contain overhang sequences at their 5` ends. The second PCR attaches sequencing adapters and unique indexes to the amplicons from the first PCR using primers with sequence complementary to the overhang sequences included in the primers for the first PCR. Table S3: Reverse transcription, PCR and index PCR reaction preparation and cycling conditions. Table S4. ccp4 and pfmget transcript abundance in samples collected from gametocyte-positive infections occurring in participants in a cohort study conducted in Malawi. [file 12936_2024_4920_MOESM1_ESM.docx]

**Additional file 1**

**Table S1.** Microhaplotypes**^a^** comprising the *pfs230* gametocyte genotyping marker.

| **Chromosome** | **Start Position** | **End Position** | **Samples^b^** | **Unique Haplotypes** | **SNPs** | **Expected Heterozygosity** |
| --- | --- | --- | --- | --- | --- | --- |
| Pf3D7_02_v3 | 375686 | 375886 | 4011 | 49 | 9 | 0.503 |
| Pf3D7_02_v3 | 375786 | 375986 | 3881 | 54 | 8 | 0.604 |
| Pf3D7_02_v3 | 375886 | 376086 | 3801 | 15 | 2 | 0.509 |

**^a^**Microhaplotypes from Tessema *et al. Journal of Infectious Diseases,* 2022 April 1; 225(7):1227-1237.

**^b^**WGS samples and accession numbers can be found in Tessema *et al.,* Supplementary Table S1

**Table S2**: RT-PCR and PCR primer sequences for *pfs230*-M3A genotyping marker with overhang adapter sequences for library preparation.

| **Primer Name** | **Overhang Adaptor Sequence (5`-3`)** |
| --- | --- |
| *pfs230*-M3A_Foward  *pfs230*-M3A_Reverse | TCG TCG GCA GCG TCA GAT GTG TAT AAG AGA CAG  GTC TCG TGG GCT CGG AGA TGT GTA TAA GAG ACA G |
|  | **Primer Sequences (5`-3`)** |
|  | CTG AAA ACG ACA ATG AAT ATG AGT  TGG GAA CAA TTG GTG GAA CA |
|  | **Full oligos (5`-3`)** |
|  | TCGTCGGCAGCGTCAGATGTGTATAAGAGACAGCTGAAAACGACAATGAATATGAGT  GTCTCGTGGGCTCGGAGATGTGTATAAGAGACAGTGGGAACAATTGGTGGAACA |
| **Primer Name** | **Primer Sequences (5`-3`)** |
| Pfs25_Forward  Pfs25_Forward | GAA ATC CCG TTT CAT ACG CTT G  AGT TTT AAC AGG ATT GCT ATC TAA |

**Figure S1:** Two-step PCR to generate amplicons for sequencing. The first PCR targets the genotyping marker sequence, and primers contain overhang sequences at their 5` ends. The second PCR attaches sequencing adapters and unique indexes to the amplicons from the first PCR using primers with sequence complementary to the overhang sequences included in the primers for the first PCR.


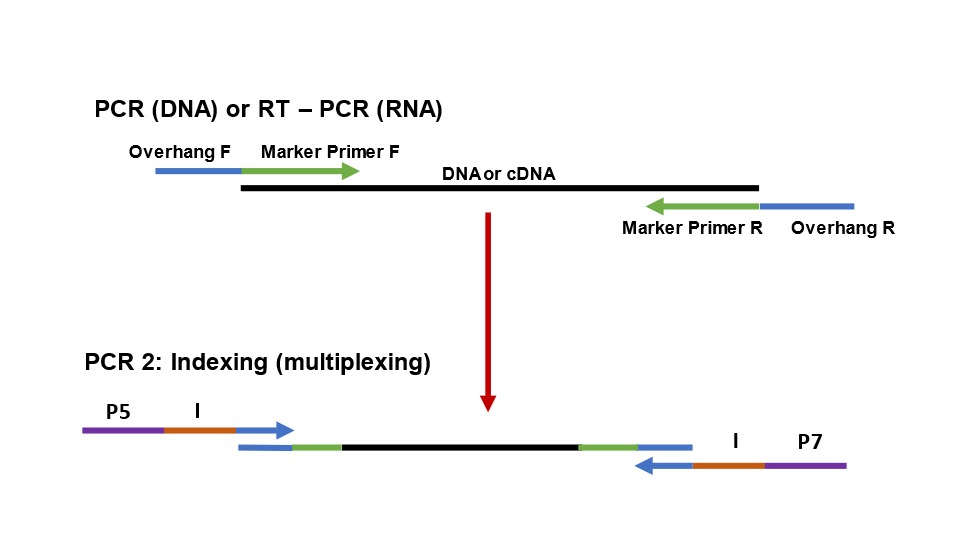


**Table S3:** Reverse transcription, PCR and index PCR reaction preparation and cycling conditions.

**Table S4.** *ccp4* and *pfmget* transcript abundance in samples collected from gametocyte-positive infections occurring in participants in a cohort study conducted in Malawi.

|  | **All gametocyte-positive infections**  **(n=594)**  **Median [IQR]** | **Successfully genotyped gametocyte-positive infections (n=209)**  **Median [IQR]** |
| --- | --- | --- |
| ***ccp4* transcripts/µL (female gametocytes)** | 0.21 [0.048 – 1.08] | 0.93 [0.048 – 3.30] |
| ***pfmget* transcripts/µL (male gametocytes)** | 0.010 [0.0020 – 0.051] | 0.046 [0.0047 – 0.16] |
| IQR: interquartile range | | |
